# Compensatory role of verbal learning and consolidation in reading and spelling of children with dyslexia

**DOI:** 10.1007/s11881-022-00264-2

**Published:** 2022-07-15

**Authors:** Robin van Rijthoven, Tijs Kleemans, Eliane Segers, Ludo Verhoeven

**Affiliations:** 1 Behavioural Science Institute, Radboud University, Nijmegen, The Netherlands; 2 OPM, Nijmegen, The Netherlands

**Keywords:** Compensation, Dyslexia, Response to intervention, Verbal consolidation, Verbal learning

## Abstract

The present study investigated the compensatory role of verbal learning and consolidation in reading and spelling of children with (*N* = 54) and without dyslexia (*N* = 36) and the role of verbal learning (learning new verbal information) and consolidation (remember the learned information over time) on the response to a phonics through spelling intervention of children with dyslexia. We also took phonological awareness, rapid automatized naming, verbal working memory, and semantics into account. Results showed that children with dyslexia performed better in verbal learning and equal in verbal consolidation compared to typically developing peers. Regression analyses revealed that verbal learning did not predict reading but did predict spelling ability, across both groups; verbal consolidation did not predict reading, nor spelling. Furthermore, neither verbal learning nor verbal consolidation was related to responsiveness to a phonics through spelling intervention in children with dyslexia. Verbal learning may thus be seen as a compensatory mechanism for spelling before the intervention for children with dyslexia but is beneficial for typically developing children as well.

## Introduction

In learning to read, children learn to integrate orthographic, phonological, and semantic codes into highly specified and redundant lexical representations (Perfetti, 2007). After having cracked the alphabetic code, they need to speed up their visual word decoding and spelling with ongoing practice (Ehri, 2005). Brain studies showed that during fluent reading, typically developing children show activation in the posterior regions, while poor readers tend to show underactivation (Paulesu et al., 2001; Shaywitz et al., 2002). This underactivation could hinder the building of an efficient connection between orthographic and phonological codes and thus can be linked to the phonological deficit that is associated with dyslexia (Göbel & Snowling, 2010; Shaywitz et al., 2003). However, poor readers may compensate for their reading and spelling problems by using their ability for verbal learning. In learning new verbal information, both the verbal learning (learning through rehearsals) and consolidation of the learned material (recall over time) are important and independent processes (Helmstaedter et al., 1997). A higher verbal learning capacity enables the learning of new phonological information which may enhance the phonological network. This, in turn, may support reading and spelling development. So far, behavioral research into this possibly compensatory role of verbal learning in children with dyslexia is limited and mostly lacking control variables and a typically reading control group. In addition, the impact of verbal learning and consolidation on responsiveness to intervention has not been tested. Therefore, in the present study, the impact of verbal learning and verbal consolidation on reading and spelling before and after a phonics intervention was examined.

### Reading and spelling in children with dyslexia

Already at a young age, children start to develop their spoken language. According to Levelt et al. (1999), this development starts with the formation of semantic representations (which word refers to which object), which are connected to phonological representations (words contain separate phonological segments). The formation of semantic and phonological representation can be seen as the foundation on which further reading and spelling development builds (Shaywitz et al., 2003). Literacy development continues when children learn to read by recoding graphemes into sounds and blend these sounds into spoken words. At the same time, children learn to spell words by dividing a spoken word into separate sounds and write the corresponding graphemes. In the beginning, children read and spell slowly. Later, reading and spelling gets faster and more fluent. According to the lexical quality hypothesis, this is the consequence of the formation of phonological, semantic, and orthographic representations and bidirectional relations between these representations during reading and spelling (Perfetti, 2007). The specificity and redundancy of the representations determine reading and spelling levels (Perfetti, 2007). Children with dyslexia are hindered in building a high-quality network by their phonological deficit; they show problems in the detection, segmentation, and manipulation of individual sounds in words (Beitchman & Young, 1997). These abilities are, at least to a large extent, dependent on the quality of the underlying phonological representations (Goswami, 2000) which can be underspecified and sometimes missing (Conrad, 2008; Göbel & Snowling, 2010). As a consequence, children may experience difficulties in reading words accurately and fluently and in spelling words correctly (Lyon et al., 2003).

Multiple neuroimaging studies found that children with dyslexia show relative underactivation in the posterior regions and relative overactivation in the anterior regions during rhyme decision tasks of both real words and pseudowords (e.g., Hoeft et al., 2007; Shaywitz et al., 2003). The posterior region is known for the integration of visual codes, phonological structures, and the phonological retrieval that enables fluent reading (Price & Friston, 1997) and has found to be associated with the phonological deficit of children with dyslexia (Brunswick et al., 1999). The anterior region is known for different aspects of language processing, making efficient processing of language possible (Bookheimer, 2002), such as the encoding of new memories, the retrieval and selection of declarative and procedural knowledge (Buckner  et al., 2001; Ullman, 2004), and for memorization of verbal information (Smith & Jonides, 1999).

Based on neuroimaging studies, Kearns et al. (2019) and Hoeft et al. (2011) suggested that children with dyslexia may use different pathways to read as compared to typically developing peers in an attempt to overcome dysfunctions in the posterior regions. The overactivation in the anterior regions could point to a compensation for the weak relationship between phonological and orthographic representations. One possible source of compensation associated with this region is the ability to learn and remember verbally presented information. Verbal learning is a way of expanding the phonological representations and thus the lexical specificity of words. As verbal learning adds new phonological information to the child’s lexicon (and therefore more specificity and redundancy), it could facilitate reading and spelling (in line with the lexical restructuring hypothesis by Metsala & Walley (2008)). Children who learn verbal information more easily may be able to form more and better specified semantic and phonological representations, which helps to recognize words while reading parts of it. Better ability to learn verbal information may thus indirectly compensate for the lack of strong and bidirectional phonological and orthographic representations. In other words, the ability to learn and remember words could expand the lexical quality as described by Perfetti (2007).

### Role of verbal learning and consolidation in reading and spelling

Throughout the years, a lot of research has been done on memory-related functions among children with dyslexia, with verbal working memory receiving most attention. Previous research showed that children with dyslexia have verbal working memory problems which are related to deficiencies in their literacy development (Kastamoniti et al., 2018). To be more specific, problems in verbal working memory lead to unstable representations and thus inefficient activation of the semantic features of words (Lukatela et al., 1998). Therefore, working memory is also related to semantic development (Gathercole, 1995). In the case of unstable phonological representations, repetition and rehearsal could lead to better outcomes as these processes stimulate the specificity of the lexicon. However, as verbal working memory itself can only store and retrieve phonological information for a limited amount of time, it has been suggested that stimulating the specificity of the lexicon itself lies not so much in the verbal working memory system, but in the storage of phonological information in long-term memory (e.g., Elbro & Jensen., 2005; Mayringer & Wimmer, 2000).

Two processes that are involved in acquiring and storing verbal information in long-term memory are verbal learning and consolidation. Verbal learning can be seen as a process of maintaining as much verbal information as possible in working memory for a short period (Kibby, 2009) with the help of repetition and rehearsal to store the verbal information properly (Van Strien, 1999). Research regarding the role of verbal learning showed that school-aged 6- to 13-year-old children with dyslexia learn verbal information less efficiently compared to controls in paired associate learning (Elbro & Jensen., 2005; Litt & Nation, 2014; Mayringer & Wimmer, 2000; Messbauer & de Jong, 2003; 2006, Wang et al., 2017) and verbal list learning (Kibby, 2009; Kramer et al., 2000; Tijms, 2004; Van Strien, 1999). Only Kibby (2009) found no differences in verbal learning ability between 9- and 13-year-old children with dyslexia and typically developing controls. Research on verbal learning tasks showed that children with dyslexia between 6 and 13 years old indeed benefit from rehearsal and external updating, although they profit less from it than controls (Elbro & Jensen, 2005; Kibby, 2009; Kramer et al., 2000; Litt & Nation, 2014; Mayringer & Wimmer, 2000; Messbauer & de Jong, 2003, 2006; Wang et al., 2017). It can be concluded that children with dyslexia may have a less efficient rehearsal and encoding mechanism which may limit the acquisition of higher quality representations of already known words (Elbro & Jensen, 2005).

After learning, new verbal material needs to be remembered over time. This is called verbal consolidation. The consolidation of this information in school-aged 8- to 13-year-old children with dyslexia is comparable with controls of the same age (Kibby, 2009; Kramer et al., 2000; Messbauer & de Jong, 2003;Van Strien, 1999) and 2 years younger reading-age controls (Elbro & Jensen, 2005). Tijms (2004) concluded that inaccurate phonological representations could interfere with the semantic processing and thus with the acquisition of verbal material. However, once a deeper semantic level is activated, no further problems appear to be encountered, and consolidation of verbal information can take place as normal. As differences were found in verbal learning and in a single case also in consolidation, these differences could be a possible cause for reading and spelling problems. However, only a few studies related verbal learning and consolidation to reading and spelling. First, some studies found that visual-verbal paired-associated learning was related to reading outcomes among typically developing children varying in age between 6 and 12 years old (Hulme et al., 2007; Litt et al., 2013; Warmington & Hulme, 2012; Windfuhr & Snowling, 2001). Second, Kibby (2009) found non-significant small partial correlations between verbal list learning and reading for 9- to 13-year-old children with dyslexia. The relations were also non-significant for typically developing children. Third, Tijms (2004) found that, among 11-year-olds with dyslexia, verbal list learning as part of a factor “phonological memory” together with digit span and interference was weakly but significantly related to reading and spelling. According to Tijms (2004), these tasks were taken together as they share the encoding of the phonological characteristics of information. More serious phonological memory deficits were found to be accompanied by weaker reading and spelling levels. It was concluded that children with dyslexia have problems in the acquisition of verbal material, but when verbal material is acquired, consolidation of verbal information takes place as normal. All studies just described operationalized verbal learning as the end result of learning (the total amount of words remembered in the last trial) instead of looking at the learning potential by means of the learning curve of each child as has been done by Kramer et al. (2000) and Van Strien (1999). Comparing all four mentioned studies, it can be concluded that differences occur in operationalization of verbal learning and age of participants. No study so far linked the learning curve as described by Van Strien (1999) and Kibby (2009) to reading and spelling outcomes.

Prior studies showed that children with dyslexia benefit most from phonics interventions for reading compared to reading fluency trainings, phonemic awareness instructions, reading comprehension trainings, auditory trainings, medical treatments, and colored overlays or lenses (Galuschka et al., 2014). For spelling, it was found that phonics, morphological, and orthographic interventions are all effective in treating spelling of children with dyslexia (Galuschka et al., 2020). Furthermore, prior studies showed that brain activation in the posterior region is more alike typically developing peers after such an intervention (Shaywitz et al., 2003; Simos et al., 2002). However, large individual differences in response to intervention were reported (Galuschka et al., 2014; 2020). Following our line of reasoning, the ability to learn and maintain verbal information could be related to variation in responsiveness. Better verbal learning and consolidation may help to develop better specified lexical representations and improve reading and spelling development.

Given the fact that dyslexia can be characterized by an underlying phonological deficit, phonological awareness, rapid automatized naming, and verbal working memory are generally considered cognitive precursor measures that predict literacy development (Shaywitz et al., 2003; Snowling, 1998). In order to determine the unique contribution of verbal learning and consolidation to reading and spelling before and after an intervention, these measures need to be included. Furthermore, variation in semantic abilities has previously been shown to impact reading (Nation & Snowling, 2004; Torppa et al., 2010) and spelling ability (Ouellette, 2010; Tainturier & Rapp, 2001) and should therefore be included as well. Out of the studies that included list learning tasks, Kibby (2009) did not include control variables, and Tijms (2004) included phonological awareness separately, and verbal working memory was included in the factor “phonological memory” together with verbal learning. Rapid automatized naming and semantics were not included. As a consequence, the unique contribution of verbal learning and consolidation is still unknown. In order to find out whether this impact is typical for children with dyslexia, control groups are necessary. However, Tijms (2004) did not include a control group, and so it is still unknown whether the reported effects are unique for children with dyslexia.

The debate on the compensatory role of verbal learning can at best be called inconclusive. First of all, verbal learning is interpreted differently across studies. The studies that related verbal learning to reading and spelling interpreted verbal learning and consolidation as a static measure in which the end result counts (e.g., Tijms, 2004), whereas both verbal learning and consolidation can be considered dynamic processes in which the learning curve is an important indicator of the ability to learn and withhold verbal information (e.g., Kramer et al., 2000). To the best of our knowledge, no prior study examined the relation between the dynamic aspect of verbal learning or consolidation and reading and spelling outcomes. Second, prior studies relating verbal learning and consolidation to reading and spelling included few or no control variables and no control group. In order to understand the unique contribution of verbal learning and verbal consolidation, studies need to include other relevant control variables (such as phonological awareness, rapid automatized naming, verbal working memory, and semantics). In order to find out if the effects of verbal learning or consolidation are typical for children with dyslexia, a control group with typically developing peers needs to be included as well. Third, no study so far examined verbal learning and consolidation in relation to response to intervention. Individual variation in the dynamics of the task might also have an impact on the intervention.

### The present study

The present study investigated the compensatory role of verbal learning and consolidation in reading and spelling of children with (*N* = 54) and without dyslexia (*N* = 36) and the role of verbal learning and consolidation on the response to a phonics through spelling intervention of children with dyslexia in order to find out whether expanding the specificity and redundancy of the (phonological) lexicon helps children with dyslexia. Verbal learning was measured by a list learning task in which children were verbally presented with a list of 15 meaningful words. Immediately after hearing the words, the children were asked to recall as many words as possible in a random order. The same list was presented five times in a row and each time the child had to name as many of the presented words as possible. Following Van Strien (1999), the result of the first trial is seen as an index of immediate memory span and the change in performance over the five trials as a measure of verbal learning. After a 30-min delay, the child was asked to name all the words they remembered. The outcome was called the verbal consolidation. We first studied the similarities and differences in verbal learning and consolidation between children with dyslexia and typically developing children. Next, we examined the relation between the individual differences in verbal learning and consolidation of both groups and their reading and spelling outcomes (before the intervention) by means of regression analyses, controlling for phonological awareness, rapid automatized naming, verbal working memory, and semantics. Furthermore, we examined the relation between verbal learning and consolidation and the response to intervention among children with dyslexia again with regression analyses and inclusion of the prementioned control variables.

In this study, we thus asked to what extent verbal learning and consolidation (a) predict reading and spelling levels in children with dyslexia compared to controls and (b) improve response to intervention in children with dyslexia. Based on the lexical quality hypothesis (see Perfetti, 2007), we expected that better verbal learning and consolidation are related to better reading and spelling outcomes, even more so for children with dyslexia (see Shaywitz et al., 2003). Furthermore, we expected that better verbal learning and consolidation are positively related to responsiveness to intervention in children with dyslexia due to the enhancement of lexical representations as well.

## Method

### Participants

Participants were children diagnosed with developmental dyslexia (37 boys, 17 girls) and typically developing children (12 boys, 24 girls)*.* All participants spoke Dutch as their first language, and all parents gave active consent to use the collected data for research purposes.

The Dutch children with dyslexia were diagnosed and received an in-service phonics through spelling intervention in a clinic for assessment and intervention for children with learning difficulties. The mean age of the children with dyslexia at the start of the assessment was 8.65 years (*SD* = 1.07). Children were in grade 2 (*n* = 12), grade 3 (*n* = 24), grade 4 (*n* = 11), grade 5 (*n* = 5), and grade 6 (*n* = 2). Ten children attended the same class an extra year. All children had semantic abilities within the normal range (mean total standardized score of four subtests forming semantics = 109.26, *SD* = 13.11).

The mean age of the children in the control group was 8.66 years (*SD* = 0.88). Children were in grade 2 (*n* = 10), grade 3 (*n* = 17), and grade 4 (*n* = 9). Two children attended the same class an extra year. All children had semantic abilities within the normal range (mean total standardized score of four subtests forming semantics = 105.00, *SD* = 14.20).

### Outcome measures

#### ***Pseudoword decoding***

Pseudoword decoding was measured with the “Klepel Test” (Van den Bos et al., 1994). Children were asked to read as many meaningless words correctly as possible within a time limit of 2 min. This task consists of 116 pseudowords presented in four rows on one sheet. The words all have a legal phonological structure of Dutch words. Words become more difficult gradually from one syllable (“taaf”) up to five syllables (“nalleroonplinteng”). Efficiency measures (i.e., total number of words read within 2 min minus number of errors) were calculated. Test scores were standardized by comparing them to norm-based peers with the help of the manual of the tests. The parallel test–retest reliability of this measure differs per age but is at least 0.89 (Van den Bos et al., 1994).

#### ***Word decoding***

Word decoding was measured with the “Brus One Minute Test” (Brus & Voeten, 2010). In this word reading fluency test, children were asked to read as many meaningful words correctly as possible within a time limit of 1 min. This task consists of 116 unrelated words presented in four rows on one sheet. Words become more difficult gradually from one syllable (“waar” [true]) up to four syllables (“tekortkoming” [shortcoming]). Efficiency measure (i.e., total number of read words minus number of errors) was calculated. Test scores were standardized by comparing them to norm-based peers with the help of the manual of the tests. The parallel test–retest reliability of this measure differs per age but is at least 0.87 (Van den Bos et al, 1994).

#### ***Spelling***

Spelling was measured with the standardized “PI word dictation” (Geelhoed & Reitsma, 1999). In this task, children were asked to write single Dutch words correctly. The dictation consisted of 135 words, divided into 9 blocks of 15 words. First a sentence including the target word was read aloud, and afterwards the target word was repeated. The test was terminated when a child failed to write at least eight out of fifteen words per block correctly. The number of correctly written words was counted. Test scores were standardized by comparing them to norm-based peers with the help of the manual of the tests. There were two versions available of the test (version A and version B). The internal consistency reliability of this measure differs per age but is at least 0.91 (Geelhoed, & Reitsma, 1999).

### Predictor measures

#### ***Verbal learning***

Verbal learning was measured by “15 words test for children” (Kingsma & van den Burg, 2005), the Dutch adaptation of the Rey Auditory Verbal Learning Test (Rey, 1941). The experimenter verbally presented the child with a list of 15 meaningful and mostly monosyllabic words. Immediately after hearing the words, the child was asked to recall as many words as possible in a random order. The same list was presented five times in a row and each time the child had to name as many of the presented words as possible. Each trial the amount of correct named words was noted. Following Van Strien (1999), the result of the first trial can be seen as an index of immediate memory span (Intercept), and the change in performance over the five trials (slope) can be seen as a measure of verbal learning. The parallel test–retest reliability of this measure is 0.70, (Van den Burg & Kingsma, 1999).

#### ***Verbal consolidation***

Verbal consolidation was measured by “15 words test for children” (Kingma and Burg, 2005), the Dutch adaptation of the Rey Auditory Verbal Learning Test (Rey, 1941). After finishing the five trials, there is a 30-min delay in which non-verbal tasks were administered. After this 30-min delay, the children were asked to name all the words they remembered, which represents the verbal consolidation. The reliability of this measure is 0.62 (Kingma and Burg, 2005).

### Control measures

#### ***Phonological awareness***

Two subtests from the “Screening Test for Dyslexia” (Kort et al., 2005a) were used. First, during “Phoneme Deletion,” children were asked to omit a phoneme from an orally presented word and speak out the remaining word (e.g., “da*k*” [roof] minus “*k*” [f] is “da” [roo]). Testing was terminated after four consecutive mistakes. Second, during the subtest “Spoonerism,” children had to switch the first sounds of two words (e.g., “*J*ohn *L*ennon” becomes “*L*ohn *J*ennon”). In both tests, all correctly formed words were counted. The test–retest reliability differs per age but is at least 0.60 (Kort et al., 2005a).

#### ***Rapid automatized naming***

RAN was measured using two subtests of “Continuous Naming and Reading Words” (Van den Bos & Lutje Spelberg, 2007). During “Naming Letters,” children had to read out loud 50 letters. During “Naming Digits,” they were asked to read out loud 50 digits. Children were asked to name these visual stimuli as quickly as possible. The time in seconds needed to finish each subtest was used for analysis, and thus a higher score expressed a weaker performance on RAN. The (split half) reliability of this measure differs per age but is at least 0.75 (Van den Bos & Lutje Spelberg, 2007).

#### ***Verbal working memory***

Verbal working memory was measured using the backward task of the Number Recall subtest from the Wechsler Intelligence Scale for Children-III (WISC-III^NL^) (Kort et al., 2005b). In this task, the experimenter pronounces sequences of digits that the child was asked to repeat in backward order. Testing was terminated after two consecutive mistakes. The number of correctly recalled sequences was counted. The (split half) reliability of this measure differs per age but is at least 0.50 (Kort et al., 2005b).

#### ***Semantics***

Semantics were measured by adding the z-scores of four subtests from the WISC-III^NL^ (Kort et al., 2005b). Based on the manual, the child received zero, one, or two points for each item. Testing was terminated after four or five (*Information*) consecutive mistakes. The (split half) reliability differs per age but is between 0.64 and 0.77 (Kort et al., 2005b). First, during “Information,” the child has to answer verbally asked questions to test their general knowledge about events, objects, places, and people. Secondly, during “Similarities,” the child has to name the similarity between two concepts. Thirdly, during “Productive vocabulary,” the experimenter pronounces a word, and the task of the child was to define the given word. Fourthly, during “Comprehension” the experimenter asked questions about social situations or common concepts. Kaufman (1975) already showed that these four measures together form a factor named “verbal comprehension,” which is also the case in the current sample (van Rijthoven et al., 2018).

### Procedure

For the purpose of this study, 99 files of Dutch children were collected from a clinic for assessment and intervention for children with learning difficulties. Due to missing data and different instruments, a sample of 54 children with Dutch as their first language remained for this study. The children were diagnosed with dyslexia following the protocol by Blomert (2006), which is in line with the definition of dyslexia of the International Dyslexia Association (2002). The Dutch protocol for diagnosis of developmental dyslexia (Blomert, 2006) states that teachers have to prove resistance to treatment and weak performances on word reading and spelling during one and a half school year. In the subsequent diagnosis, a phonological deficit needs to be evidenced, and other explanations of reading or spelling problems need to be excluded by a certified clinical psychologist. After assessment all children diagnosed with dyslexia received an in-service phonic through spelling intervention in a clinic for assessment and intervention for children with learning difficulties. All children were tested between 2009 and 2013 in two consecutive mornings by clinicians on above mentioned measures. For semantics and verbal working memory, a few raw scores were missing (2–4 per variable). Furthermore, spelling dictation of one child was missing. Two or three weeks after the assessment, the phonics through spelling intervention started for 52 children. After the intervention, all participants received the posttest, including pseudoword decoding, word decoding, and spelling measures. Eight children were not tested for pseudoword decoding at posttest.

For the current study, data from a control group was included. Data from the control group was gathered at a primary school in the east of the Netherlands. Parents were informed about the purpose of the study by means of a letter and flyer with a link to an online consent form. In the flyer, they were told we were hoping to get permission to test the verbal learning ability and reading and spelling levels along with some control variables in order to study differences and similarities between children with dyslexia and typically developing children in their abilities and the relation between these abilities. In total 36 parents gave permission for their child to participate. Children from the control group were tested by graduate students, during two mornings within 2 weeks. The graduate students received a training by one of the authors and all the necessary material in advance. Tests were performed in two blocks (Block A and Block B). The blocks were randomized over the two days. However, the test order within the blocks was fixed. Block A included measures for spelling, verbal learning, word reading, pseudoword reading, rapid automatized naming, and verbal consolidation. Block B included the measures for phonological awareness and the five subtests of the WISC-III^NL^. Both blocks lasted about 45 min and took place in a quiet room within the school of the children. Children were picked up from the classroom for participation. All outcome variables were standardized by comparing them to norm-based peers with help of the manual of the tests. The raw scores (total amount of words correctly written or read) were converted into percentile scores (a score of for instance 35 meant that 35% of the children scored at or below the child in question). A higher score meant a better performance on the task. For the predictor measures, z-scores (based on raw scores) were used to standardize all measures. For phonological awareness, rapid automatized naming, and semantics, a composite score of these z-scores was computed.

### Phonics through spelling intervention

A phonics through spelling intervention aims to reach a functional level of technical reading and spelling by means of combining reading and writing in one intervention following the protocol by Blomert (2006). Unique to a phonics through spelling intervention is that 50% of the amount of time available during the intervention is spend on spelling, while most studies include less or sometimes no word spelling. Children had a weekly 45-min session with a clinician. The mean length of the intervention was 27.27 weeks (SD = 4.88). Variation in the length of the intervention occurred due to variation in the post-intervention assessment schedule (for instance due to holidays or personal circumstances). Furthermore, variation in the length of the intervention occurred due to variation in time needed to acquire 80% accuracy levels and improved fluency levels as described below. During the sessions, the clinician tailored the intervention as much as possible to each child’s needs. Explicit direct instruction, guided exercises, and feedback were given according to each child’s needs. The continuity of quality during assessment and intervention was guaranteed by supervision of certified clinical health psychologists. The intervention included two stages:

1. Phonological spelling

The intervention started with practice of the phonological base of reading and spelling due to learning the grapheme-phoneme-correspondences (GPC). After learning the GPCs, children learned to use this letter knowledge in reading and writing words and sentences/texts by using an explicit strategy. When children mastered the basic levels, children learned to read and write words based on syllables as well. Accuracy was trained first, followed by efficiency and words and sentences/texts increased in difficulty. Feedback was given on accuracy and later also on efficiency.

2) Orthographic spelling

Dutch is a rather transparent language, but still morphological rules and orthographic patterns need to be learned to write and read words (mostly polysyllabic words) correctly. The morphological rules and orthographic patterns were taught according to each child’s needs.

In order to rehearse the above-mentioned spelling and reading knowledge, children had to do home exercises for reading and spelling. Parents were asked to train four times a week during 15 min with prescribed exercises. All parents have confirmed that this has been complied with. Parents reflected on the home exercises in a day-to-day logbook. When a child reached an accuracy of 80% during practice (read or write 80% of the words correctly) and improved significant in their fluency (more fluent compared to the first time words were read), the clinician moved on to the next topic of intervention. This formative testing was sustained throughout the entire intervention. Therefore, variation in the length of the program is present.

### Analytic approach

By means of regression analyses, intercept and slope of the five trials of the 15 words test were calculated for each participant to quantify verbal learning. To check whether groups differed on verbal learning and consolidation, two repeated measures ANOVAs were conducted. To answer the research question and maintain enough statistical power, a total of twelve separate hierarchical regressions were conducted, four for each outcome measure (i.e., word decoding, pseudoword decoding, and spelling). The first and second hierarchical regression tested the effect of verbal learning on the outcome measure before and after the intervention, and the third and fourth hierarchical regression tested the effect of verbal consolidation on the outcome measures before and after the intervention. In all cases, relations were tested without control variables, and first and later control variables were added separately. Based on the fact that verbal learning and verbal consolidation are separate processes (Helmstaedter et al., 1997), we assumed that analyzing the effects of these factors separately would be a good way to maintain enough power as in the case of the response to intervention only 52 participants were present. In line with Harris (1985), who reported that analysis should include an absolute minimum of 10 participants per predictor, this meant we could include five predictors at most. In order to express the response to intervention, the individual mean change between pre- and posttest (with percentile scores) was calculated by subtracting pre- from posttest scores. Following Gollwitzer et al., (2014), change scores are reliable when two requirements are met. First, standard deviation must differ between measurement occasions. Second, there needs to be a non-zero variation in observed difference scores in order to define the reliability. In order to rule out the effects of variation in length of the intervention, the individual change score was divided by the number of sessions the intervention for each individual lasted. Working with change scores implied that the differences in individual pretest scores were not taken into account. Pretest scores were included as a control variable to control for variation.

## Results

Descriptive statistics for all measures are presented in Table 1. To compare children with dyslexia and typically developing children, we performed *t-*tests for independent samples with Holm-Bonferroni correction (Holm, 1979). Children with dyslexia scored below typically developing children on all outcome measures and on predictor measures phonological awareness measures (except the raw scores of phoneme deletion) and rapid naming measures (except the raw scores of letter naming). No differences were found in verbal working memory or semantics. Regarding verbal learning, t-tests showed no group differences at trial 1, but significant differences at trials 2 to 5, with lower scores for the typically developing children. The total score over five trials differed, but the scaled score, intercept, and slope over five trials did not differ significantly. Finally, no differences were found regarding verbal consolidation. Correlations between all used measures are presented in Table 2.

**Table 1:** Descriptive statistics for children with dyslexia at pretest and typically developing children

		Children with dyslexia		Typically developing children		Holm-Bonferroni corrected t-tests	
		*M*	*SD*	*M*	*SD*	*t*	*d*
Outcome measures							
Word decoding	Raw scores	31.37	13.23	51.94	18.69	− 5.72***	1.27
	Percentiles	5.51	5.61	45.06	33.43	− 7.03***	1.65
Pseudoword decoding	Raw scores	20.61	10.05	46.11	21.42	− 6.67***	1.52
	Percentiles	8.39	8.09	51.86	31.33	− 8.15***	1.90
Spelling	Raw scores	49.96	28.31	72.97	24.38	− 3.98**	0.90
	Percentiles	7.74	14.50	62.11	33.88	− 9.08***	2.06
Predictor measures							
Phonological awareness							
Spoonerism	Raw scores	3.07	3.27	6.42	3.78	− 4.47***	0.95
	Scaled scores	7.80	2.13	10.25	2.90	− 4.35**	0.96
Phoneme deletion	Raw scores	8.02	2.38	9.50	2.04	− 3.07	0.67
	Scaled scores	7.67	2.33	9.89	2.72	− 4.14**	0.88
Rapid automatized naming							
Letter naming	Raw scores	39.85	9.25	35.67	10.93	1.96	0.41
	Scaled scores	5.43	2.32	8.44	3.73	− 4.33**	0.97
Digit naming	Raw scores	36.26	10.00	29.14	5.68	4.23**	0.88
	Scaled scores	6.33	2.89	9.94	3.11	− 5.64***	1.20
Verbal working memory							
Digit span backwards	Raw scores	4.14	1.37	5.42	2.61	− 2.68	0.61
Semantics							
Information	Raw scores	13.35	3.84	12.44	2.32	1.37	0.29
	Scaled scores	10.78	2.40	10.89	2.33	− 0.22	0.05
Similarities	Raw scores	14.60	5.14	11.89	3.85	2.82	0.60
	Scaled scores	12.30	3.14	10.89	3.16	2.08	0.45
Vocabulary	Raw scores	31.44	7.14	28.22	6.58	2.15	0.60
	Scaled scores	11.56	2.51	10.22	2.89	1.11	0.24
Comprehension	Raw scores	20.94	5.88	18.00	4.65	2.51	0.55
	Scaled scores	11.54	2.57	10.50	2.50	1.90	0.41
Verbal learning							
Trial 1	Raw scores	4.74	1.39	4.67	1.33	0.25	0.05
Trial 2	Raw scores	7.41	2.02	6.06	1.94	3.16*	0.68
Trial 3	Raw scores	8.59	1.95	7.08	2.09	3.50*	0.75
Trial 4	Raw scores	9.54	2.08	7.83	2.59	3.45*	0.73
Trial 5	Raw scores	10.35	1.96	8.53	2.60	3.79**	0.79
Total score over five trials	Raw scores	40.63	7.49	34.17	8.47	3.80**	0.81
	Scaled scores	4.83	2.81	3.61	2.50	2.11	0.46
Intercept trial 1–5	Based on raw scores	5.46	1.51	4.93	1.55	1.59	0.35
Slope trial 1–5	Based on raw scores	1.34	0.48	1.53	3.73	− 0.38	0.07
Verbal consolidation							
Delayed recall (trial 6)	Raw scores	8.50	2.34	7.92	2.60	1.11	0.23
	Scaled scores based on age	4.56	2.48	4.14	2.52	0.78	0.16
	Scaled scores based on total score	4.69	2.88	5.42	2.87	− 1.18	0.25

^*^p < .05. **p < .01. ***p < .001

**Table 2:** Pearson correlations between the outcome measures and predictor. Below the diagonal, children with dyslexia; above the diagonal, typically developing children

	1	2	3	4	5	6	7	8	9	10	11	12	13	14
1. Word decoding	-	.923**	.749**	.496**	− .326	.261	.380*	.398*	− .260	− .272	− .205	-	-	-
2. Pseudoword decoding	.592**	-	.649**	.473**	− .409**	.266	.374*	.321	− .314	− .263	− .202	-	-	-
3. Spelling	− .069	.137	-	.310	− .099	.329	.265	.340*	.070	− .100	.033	-	-	-
4. Phonological awareness	.202	.221	.211	-	− .085	.180	.464**	.101	− .150	− .093	− .113	-	-	-
5. Ran	− .402**	− .250	.004	− .279*	-	− .069	− .379*	− .350*	.234	.109	− .047	-	-	-
6. Verbal working memory	.007	.167	.432**	.160	.133	-	.237	.024	− .071	− .252	.081	-	-	-
7. Semantics	.152	.150	.054	.392**	− .363**	.101	-	.384*	− .146	.093	.234	-	-	-
8. Intercept 15wt	.550	− .034	.081	.281*	− .184	.122	.315*	-	− .420*	.140	.234	-	-	-
9. Slope 15wt	− .115	− .075	.058	.149	− .066	− .090	.095	− .327*	-	.363*	.236	-	-	-
10. Trial 5	− .014	− .068	.057	.252	− .280*	.022	.350*	.379**	.668**	-	.741**	-		
11. Verbal consolidation (trial 6)	.157	.077	.055	.202	− .367**	.097	.450**	.551**	.135	.557**	-	-	-	-
12. Word decoding change per session	.065	.195	.267	.123	− .195	.196	.034	.058	− .043	− .011	− .031	-	-	-
13. Pseudoword decoding change per session	.161	− .107	.018	− .074	− .147	.084	− .161	.015	− .031	.044	.128	.344*	-	-
14. Spelling change per session	.304*	.251	− .330*	.357*	− .188	.035	.450**	.174	− .034	.069	.272	− .122	− .154	-

^*^p < .05. **p < .01. ***p < .001

Note. The change per session is calculated by subtracting pre- from posttest scores and dividing the outcome by the number of sessions the intervention lasted

### Differences in verbal learning and consolidation

Before answering the research question, we checked for group differences by conducting two general linear model repeated measure analyses. Firstly, we studied the difference between children with dyslexia and typically developing children in their verbal learning over the five trials. Verbal learning was within subject factor (the five consecutive trials), and group (children with dyslexia versus typically developing children) was the between subjects’ factor. Mauchly’s test indicated that the assumption of sphericity had been violated for the main effects of verbal learning, χ^2^ (9) = 25.167, *p* = 0.003. Therefore, degrees of freedom were corrected using Huyn-Feldt estimates of sphericity (*ε* = 0.85 for verbal learning). Results showed that there was a main effect of verbal learning, *F*(3.65, 320.77) = 154.136, *p* < 0.001, η^2^_p_ = 0.637, Group *F*(1, 88) = 14.468, *p* < 0.001, η^2^_p_ = 0.141, as well as an interaction between verbal learning and group, *F*(3.645, 320.77) = 5.592, *p* < 0.001, η^2^_p_ = 0.060. While based on the Holm-Bonferroni corrected t-test (see Table 1) the groups did not differ at T1 (*p* = 0.802), the Holm-Bonferroni corrected t-test showed significant differences at all subsequent trials (*p* = 0.002), due to a higher growth for the group with dyslexia between T1 and T2 (*p* < 0.001).

**Fig. 1:**
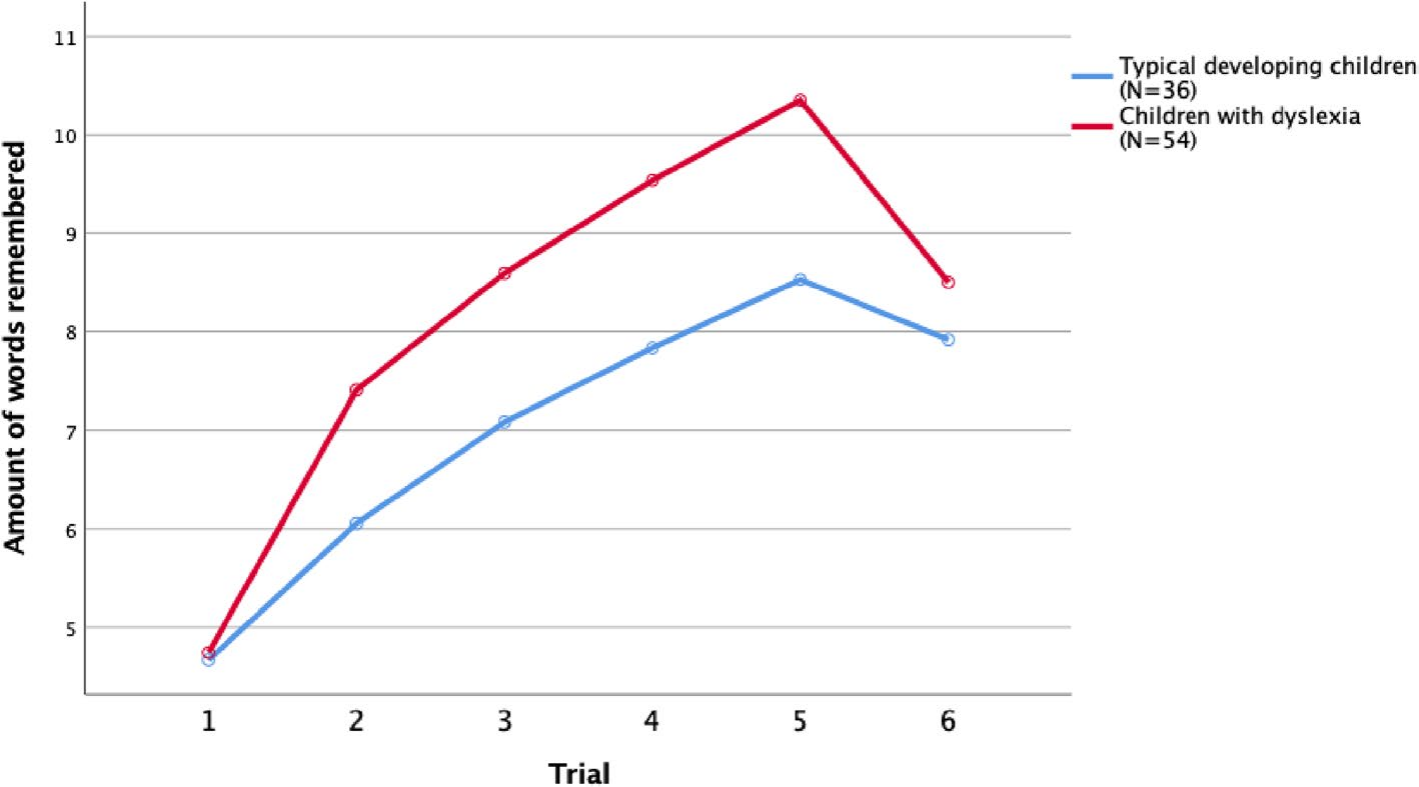
Group average words remembered from trials 1–5 and delayed recall for children with dyslexia (*N* = 54) and typically developing children (*N* = 36)

Secondly, we studied the difference between children with dyslexia and typically developing children in verbal consolidation (the amount of words remembered at T6). The amount of words remembered was the within subject’s factor (fifth trial and verbal consolidation/T6), and group (children with dyslexia versus typically developing children) was the between subjects’ factor. There was a main effect of words remembered, *F*(1, 88) = 33.386, *p* < 0.001, η^2^_p_ = 0.275, a main effect of Group *F*(1, 88) = 6.930, *p* = 0.010, η^2^_p_ = 0.073, as well as an interaction words remembered × group, *F*(1, 88) = 8.473, *p* = 0.005, η^2^_p_ = 0.088. All children forgot words over time, but children with dyslexia forgot more words compared to typically developing children. Based on the Holm-Bonferroni corrected t-test (see Table 1), children with dyslexia seem to remember significantly more words at trial 5 compared to the typically developing children (*p* < 0.001), and at the moment of verbal consolidation (i.e., T6), there seems to be no difference in the amount of remembered words over time (*p* = 0.271). Results are depicted in Fig. 1.

### Predicting reading and spelling by verbal learning and consolidation

To answer the first part of the research question, regarding the effect of verbal learning and consolidation on reading and spelling outcomes before the intervention, six hierarchical regression analyses were conducted. Three hierarchical regression analyses were performed to test the effect of verbal learning on word decoding, pseudoword decoding, and spelling followed by three hierarchical regression analysis to test the effect of verbal consolidation using the same outcome measures. In the first set of three hierarchical regression analyses, group was entered in step 1 (typically developing children = 0, children with dyslexia = 1), followed by the intercept and the slope of verbal learning (trials 1–5) in step 2. In step 3, finally, the corresponding interaction terms between group and the predictor measures were included to test whether the effects of the predictors are stronger for one of both groups. In the second set of three hierarchical regression analyses, trial 5 and verbal consolidation were entered in step 2.

The results (see Table 3) showed that group was a significant predictor for all three outcome measures. Children with dyslexia read and spell less words correctly (step 1). Adding step 2 to the model resulted in a significant improvement for all three dependent variables. In step 2, group again predicted all outcome measures. Furthermore, slope predicted pseudoword decoding, and intercept predicted spelling: a less steep slope indicated higher pseudoword decoding scores, and a higher intercept indicated higher spelling scores. Better verbal learning appears to associate with lower pseudoword decoding scores. A higher immediate memory span appears to result in better spelling skills. Step 3 was a significant improvement of the model for spelling, but not for word and pseudoword decoding. Intercept, slope, and the group × intercept interaction were all significant predictors of spelling. A steeper slope and a higher intercept were related to higher spelling scores. The effect of the intercept is larger for one of both groups. To be more precise, as can be seen in Table 2, especially for typically developing children, a higher intercept is related to higher spelling scores. This shows that typically developing children seem to benefit more from their immediate memory span capacity in learning to spell compared to children with dyslexia.

**Table 3:** The role of verbal learning (e.g., slope T1–T5) in predicting percentile scores of word decoding, pseudoword decoding, and spelling pretest in children with dyslexia and typically developing children, controlled for immediate recall, and Cohen’s f^2^

		Word decoding pretest				Pseudoword decoding pretest				Spelling pretest			
			*ΔR*^*2*^	*f*^*2*^	*β*		*ΔR*^*2*^	*f*^*2*^	*β*		*ΔR*^*2*^	*f*^*2*^	*β*
Step 1			.453***	0.828			.519***	1.079			.554***	1.242	
	Group				− 0.673***				− 0.720***				− 0.744***
Step 2			.056**	0.114			.048*	0.111			.031*	0.072	
	Group				− 0.704***				− 0.740***				− 0.771***
	Intercept (T1–T5)				0.152				0.075				0.181*
	Slope (T1–T5)				− 0.143				− 0.185*				0.098
Step 3			.033	0.072			.015	0.036			.031*	0.081	
	Group				− 0.055				− 0.317				− 0.165
	Intercept (T1–T5)				0.403**				0.241				0.421**
	Slope (T1–T5)				− 0.082				− 0.146				0.155*
	Group × intercept (T1–T5)				− 0.747*				− 0.494				− 0.714*
	Group × slope (T1–T5)				− 0.004				0.005				0.012
Total *R*^2^_adj_		.515				.557				.592*			

^*^p < .05. **p < .01. ***p < .001

In order to check if the found effects remain present when controlling for the severity of the reading and spelling problems (phonological awareness, rapid naming, and verbal working memory) and semantic abilities, these variables were added separately in steps 2 and 3 of each hierarchical regression analyses. The effect of slope on pseudoword decoding and the effect of intercept on spelling remained significant in step 2 for each predictive variable which indicates that despite the severity of the reading and spelling problems or the semantic abilities of children, the higher verbal learning rates still predict lower scores in pseudoword decoding and that a higher immediate memory span still predicts higher spelling scores. Step 3 was no longer a significant improvement when adding these variables with the exception of phonological awareness. When adding phonological awareness, step 3 became significant for word decoding and pseudoword decoding as well, showing significant effects of intercept and the group × intercept interaction on word decoding and no significant effects of intercept or slope for pseudoword decoding. The immediate memory span appeared to be associated with word reading and spelling performances for all children but even more so for typically developing children.

The results of the second set of three hierarchical regression analyses, regarding the effect of trial 5 and verbal consolidation (see Table 4) showed that group was a significant predictor for all three outcome measures (step 1). Children with dyslexia read and spell less words correctly (step 1). Step 2 and step 3 were both no significant improvement of the model. As a result, trial 5 and verbal consolidation were both not significantly associated to reading and spelling after controlling for group.

**Table 4:** The role of verbal consolidation (e.g., trial 6) in predicting percentile scores of word decoding, pseudoword decoding, and spelling pretest in children with dyslexia and typically developing children, controlled for trial 5 (e.g., intercept), and Cohen’s f^2^

		Word decoding pretest				Pseudoword decoding pretest				Spelling pretest			
			*ΔR*^*2*^	*f*^*2*^	*β*		*ΔR*^*2*^	*f*^*2*^	*β*		*ΔR*^*2*^	*f*^*2*^	*β*
Step 1			.453***	0.828			.519***	1.079			.554***	1.242	
	Group				− .673***				− 0.720***				− 0.744***
Step 2			.022	0.042			.019	0.041			.005	0.009	
	Group				− .611***				− 0.662***				− 0.718***
	Intercept (trial 5)				− .171				− 0.161				− 0.093
	Verbal consolidation (trial 6)				.020				0.019				0.082
Step 3			.018	0.036			.012	0.027			.008	0.018	
	Group				− 1.193***				− 1.141***				− 0.893**
	Intercept (trial 5)				− .284				− 0.245				− 0.241
	Verbal consolidation (trial 6)				− .008				− 0.016				0.210
	Group × intercept (trial 5)				.552				0.423				0.575
	Group × verbal consolidation (trial 6)				.106				0.118				− 0.364
Total *R*^2^_adj_		.463				.524				.540			

^*^p < .05. **p < .01. ***p < .001

Finally, it is important to mention that when we added phonological awareness, rapid automatized naming, verbal working memory, or semantics separately in step 2, combined with a corresponding interaction term with group in step 3, the effects of trial 5 and verbal consolidation did not change and remained non-significant.

### Predicting response to intervention by verbal learning and consolidation

To answer the second part of the research question, regarding the impact of verbal learning and consolidation on the response to a phonics-through-spelling intervention, we again conducted six hierarchical regression analyses. The same procedure was followed as in predicting reading and spelling outcomes, but with different outcome measures (i.e., change per session for word decoding, pseudoword decoding, and spelling). The descriptive statistics of these outcome measures are presented in Table 5. In the analyses, pretest was included as a control variable. The results (see Table 6 and 7) showed an effect of pretest on spelling, but no significant effects of verbal learning or consolidation on each of the outcome measures: verbal learning and consolidation were not significantly linked to progress made in a phonics through spelling intervention.

**Table 5:** Descriptive statistics of outcome measures (raw and percentile) including pre- and posttest scores as well as change scores and change per session

	Raw scores		Percentile scores	
	*M*	*SD*	*M*	*SD*
**Word decoding**				
Pretest	31.37	13.23	5.51	5.61
Posttest	41.04	14.74	9.70	11.20
Change scores	10.29	6.35	4.16	9.18
Change per session	0.38	.23	0.15	0.34
**Pseudoword decoding**				
Pretest	20.61	10.05	8.39	8.09
Posttest	29.36	12.28	12.63	11.74
Change scores	9.59	9.05	4.36	8.92
Change per session	0.38	0.34	0.18	0.34
**Spelling**				
Pretest	49.96	28.30	7.74	14.50
Posttest	75.20	27.47	22.00	20.54
Change scores	27.39	11.44	8.71	17.36
Change per session	1.02	0.43	0.30	0.62

Note. The change scores are calculated by subtracting pre- from posttest scores. The change per session was calculated by dividing the change scores by the number of sessions the intervention lasted

**Table 6:** The role of verbal learning (e.g., slope T1–T5) in predicting change per session of word decoding, pseudoword decoding, and spelling posttest in children with dyslexia, controlled for immediate recall (e.g., intercept T1–T5), and Cohen’s f.^2^

		Change per session word decoding				Change per session pseudoword decoding				Change per session spelling			
			*ΔR*^*2*^	*f*^*2*^	*β*		*ΔR*^*2*^	*f*^*2*^	*β*		*ΔR*^*2*^	*f*^*2*^	*β*
Step 1			.004	0.004			.001*	0.001			.031	0.032	
	Intercept (T1–T5)				0.049				− 0.007				0.182
	Slope (T1–T5)				− 0.027				− 0.029				0.025
Step 2			.003	0.003			.012	0.012			.100*	0.516	
	Intercept (T1–T5)				0.046				< 0.001				0.154
	Slope (T1–T5)				− 0.021				− 0.041				0.023
	Pretest				0.059				0.111				− 0.317*
Total *R*^2^_adj_		-.055				-.061				− .075*			

^*^p < .05. **p < .01. ***p < .001

**Table 7:** The role of verbal consolidation (e.g., trial 6) in predicting change per session of word decoding, pseudoword decoding, and spelling posttest in children with dyslexia, controlled for trial 5 (e.g., intercept), and Cohen’s f.^2^

		Change per session word decoding				Change per session pseudoword decoding				Change per session spelling			
			*ΔR*^*2*^	*f*^*2*^	*β*		*ΔR*^*2*^	*f*^*2*^	*β*		*ΔR*^*2*^	*f*^*2*^	*β*
Step 1			.001	0.001			.018	0.018			.087	0.095	
	Intercept (trial 5)				0.009				− 0.050				− 0.139
	Verbal consolidation (trial 6)				− 0.36				0.158				0.354*
Step 2			.005	0.005			.013	0.013			.070	0.083	
	Intercept (trial 5)				0.021				− 0.067				− 0.121
	Verbal consolidation (trial 6)				− 0.056				0.170				0.275
	Pretest				0.075				− 0.116				− 0.274
Total *R*^2^_adj_		− .056				− .041				.103			

^*^p < .05. **p < .01. ***p < .001

## Discussion

The present study investigated the extent to which verbal learning and consolidation (a) predict reading and spelling levels in children with dyslexia compared to controls and (b) improve response to intervention in children with dyslexia. It was found that verbal learning was not related to reading, whereas it was related to spelling. Furthermore, verbal consolidation was found not to be related to reading nor spelling outcomes. Immediate memory span was related to word decoding when controlling for phonological awareness, and it was positively related to spelling skills notwithstanding individual differences in the included cognitive factors, i.e., phonological awareness, rapid automatized naming, verbal working memory, and semantics. The influence of the immediate memory span (amount of words remembered in the first trial) on word decoding and spelling was found for all children but was even stronger for typically developing children. No compensatory role of verbal consolidation on spelling was found, and both verbal learning and consolidation did not improve the response to a phonics through spelling intervention.

Prior to answering our research questions, we compared verbal learning and consolidation of children with dyslexia and their typically developing controls. Both groups performed equally on the immediate memory span. Children with dyslexia performed better in verbal learning, but they remembered less words correctly over time compared to typically developing children. The verbal consolidation (words remembered at trial 6) was equal to typically developing children as children with dyslexia forgot more words over time between trial 5 and 6. In line with Kramer et al., (2000), both groups started by remembering the same amount of words after the first learning trial (immediate memory span). In our study, children with dyslexia with a mean age of 8.65 years outperformed typically developing children in verbal learning during subsequent trials (trials 2–5), whereas Kramer et al., (2000) and Van Strien (1999) found typically developing children around 8- to 13-years-old to outperform children with dyslexia, and Kibby, (2009) found no differences among children aged 9 to 13 years old. Our finding that children with dyslexia declined more over time than typically developing children contrasts previous findings of similar declines in words remembered by both groups (e.g., Kibby, 2009). This could have multiple explanations. Firstly, it could be due to the fact that children with dyslexia in our study were slightly younger than in previous studies on list learning. Younger children practice word attack strategies like rehearsal of words excessively in school, and especially children with dyslexia need these strategies as they have to update information constantly due to their problems in verbal working memory (Puolakanaho et al., 2007). Since children with dyslexia rely more on alternative pathways to learn to read (e.g., Kearns et al., 2019), verbal learning could be a source of compensation for younger children with dyslexia. Secondly, differences between studies could also indicate that differences between the two groups were rather small and therefore somewhat unstable. Thirdly, different learning strategies that were taught to children in their schools could explain the contradicting results.

Our first research question was whether verbal learning and consolidation predict reading and spelling in children with dyslexia compared to controls. We expected verbal learning and consolidation to be associated with reading and spelling outcomes, even more so in children with dyslexia (see Shaywitz et al., 2003). We found that verbal learning was not related to word reading, negatively related to pseudoword reading for children with dyslexia (this negative relation disappeared when controlling for phonological awareness), and positively related to spelling for both children with dyslexia and typically developing children. The effects were small to medium. Furthermore, no compensatory role of verbal consolidation was found.

The finding that verbal learning was negatively related to pseudoword reading could indicate that especially children with decoding problems train and use their verbal learning ability in an attempt to overcome these problems. Pseudoword reading is in the end a task that requires decoding skills mostly. The fact that this negative effect disappeared when we added phonological awareness to the model indicates that these decoding difficulties are phonologically based. The fact that verbal learning did not positively influence children’s reading contrasts findings by Tijms (2004), but the positive influence of verbal learning on spelling levels among children with dyslexia is in line with findings by Tijms (2004). In addition, we showed the unique influence of verbal learning on spelling in both children with dyslexia and typically developing peers. Overall, the results fit the idea that better verbal learning may support the specificity and redundancy of the phonological lexicon and therefore stimulates literacy development (Shaywitz et al., 2003) but shows that the effect is limited to spelling only. This can be explained by the fact that spelling relies more on phonological representations compared to reading (Landerl & Wimmer, 2008). This outcome seems to be in line with the statistical-learning perspective on spelling development, which emphasizes that the spelling of children reflects the input to which children have been exposed. This input is filtered through their learning mechanisms (Pollo et al., 2007). Children with stronger verbal learning abilities (their learning mechanism) can learn more from exposure to verbal input, which benefits their spelling.

The results showed no compensatory role of verbal consolidation. This is in line with the conclusion of Tijms (2004) who concluded that once a deeper semantic level is activated, no further problems appear to be encountered, and consolidation of verbal information can take place as normal. It could also be due to the fact that verbal consolidation is closely related to automaticity (Manoach & Stickgold, 2009). Indeed, in our study, we found rapid automatized naming to be associated to verbal consolidation for children with dyslexia. It is interesting to note that the above described results hold even when phonological awareness, rapid automatized naming, verbal working memory, and semantics are included in the analysis. Only the addition of phonological awareness changed the results slightly, as the negative effect of verbal learning on pseudoword decoding was no longer significant and immediate memory span appeared to predict word reading.

The second research question was on the compensatory role of verbal learning and consolidation on response to intervention among children with dyslexia. While we expected that better verbal learning and consolidation would be positively related to responsiveness to intervention in children with dyslexia due to the enhancement of semantic and phonological representations, we did not find such a relation. After intervention, children are better able to integrate visual codes, phonological structures, and the phonological retrieval which enables them to read more fluently (Price & Friston, 1997; Simos et al., 2002). We may thus speculate that a tailored intervention diminishes the need for compensation for weaker spelling abilities via verbal learning.

This study adds to already existing literature by relating the dynamic process of verbal learning and consolidation to reading and spelling outcomes of children with and without dyslexia and study the impact on response to intervention as well. Some limitations should be acknowledged at this point along with directions of future research. Firstly, although we used a standardized Dutch test for verbal learning and consolidation, this measure of verbal learning contains high-frequency words and could, therefore, also be interpreted as the reactivation of word representations clustered together instead of learning new words and withhold this cluster instead of actual consolidation of new words. Therefore, it would be recommended to include tasks in which new words need to be learned as well (e.g., Elbro & Jensen, 2005). Second, we followed the children with dyslexia over time without incorporating a (randomized) control group to evaluate the effects of the intervention. The latter group could be included in future research in order to find out if within the typically developing group verbal learning and consolidation does not influence the change due to intervention as well. Third, we did not control for the socio-economic status or parental educational level. As prior studies showed that socio-economic status of parents predicts semantic growth (Romeo et al., 2018), this could have influenced our results. We recommend future research to include this variable in their analyses. Fourth, control measures can be refined by choosing a verbal working memory test with higher reliability and by adding a measure for morphological awareness which is also addressed in the intervention. Fifth, differences in verbal learning could also be related to school-related factors such as the learning strategies that children are being taught at different schools. Future research could include schoolbound learning strategies in order to rule out the possibility that this caused the found differences. Finally, this study included a rather small sample. Although we calculated a priori that we should have enough statistical power to conduct the analyses, effect sizes in terms of Cohen’s f^2^ in the regression analyses were relatively small. Therefore, the results should be interpreted with caution. Follow-up studies with larger sample sizes are recommended.

To conclude, the present findings show that verbal learning is positively related to spelling skills for all children. Furthermore, the immediate memory span had a positive influence on spelling and word decoding of children with dyslexia and even more so for their typically developing peers. Moreover, both verbal learning and consolidation were not related to reading and spelling outcomes after a phonics through spelling intervention. The present findings also show that even before an intervention, verbal learning may facilitate spelling. Therefore, it can be recommended to monitor the verbal learning capacity of all children and not just children with dyslexia during their early spelling development. In a similar vein, it is evidenced that, since younger children seem to rely on their phonological and semantic representations, spelling strategies like oral word repeating also may benefit spelling development.
